# Protozoans in subgingival biofilm: clinical and bacterial associated factors and impact of scaling and root planing treatment

**DOI:** 10.1080/20002297.2019.1693222

**Published:** 2019-11-25

**Authors:** Marie Dubar, Marie-Laure Zaffino, Thomas Remen, Nathalie Thilly, Lisiane Cunat, Marie-Claire Machouart, Catherine Bisson

**Affiliations:** a Department of Periodontology, School of Dentistry, Lille University Hospital, Lille, France; b Laboratory Stress, Immunity, University of Lorraine, Nancy, France; c Platform Support for Clinical Research, University Hospital of Nancy, Nancy, France; d Laboratory of Parasitology - Mycology, University Hospital of Nancy, Vandoeuvre-Lès-Nancy, France; e Department of Periodontology, University Hospital and University of Lorraine, Nancy, France

**Keywords:** *Trichomonas tenax*, *Entamoeba gingivalis*, periodontitis, periodontopathogens, clinical periodontal parameters, subgingival microbiota, scaling and root planing

## Abstract

**Objective**: In patients with periodontitis, identification of protozoans and evaluation of some bacteria and clinical parameters associated and assessment of scaling and root planing (SRP) impact on their detection.

**Methods**: Before and after SRP, subgingival microbiota was collected in two pathological and one healthy site from 30 periodontitis patients. One healthy site from 30 control patients was also sampled. The usual clinical periodontal parameters were recorded; microbial detection was determined by PCR hybridization system for bacteria and qPCR for protozoans.

**Results**: In periodontitis group, *Trichomonas tenax* and two subtypes of *Entamoeba gingivalis* (ST1 and a variant ST2) were detected in respectively 33.3%, 70% and 18.3% of pathological samples, and in 6.7%, 10% and 3.3% healthy samples. In control group, ST1 alone was found in 3.3% of individuals. ST1 was associated with Gingival Index, Clinical Attachment Level (p ≤ 0.03) and with the total bacterial count (p = 0.02). *T. tenax* alone was associated with *P. gingivalis, T. denticola* and *E. nodatum* (p ≤ 0,02). After therapy, only *T. tenax* detection decreased significantly (p = 0.004) and no association between the protozoan elimination and improvement of pathological sites was found.

**Conclusions**: Protozoans were associated with some clinical parameters and/or periodontopathogens in patients with periodontitis.

## Introduction

Periodontitis is one of the most common human pathologies contributing to the global burden of chronic disease. This pathology is a public health problem because it is widespread (more than 50% of the adult population are affected) and impairs the oral and general well-being of people. Periodontitis has both a local and systemic impact. Periodontal disease is the result of the degradation of the tissue supporting the teeth; it is caused by certain opportunistic bacteria from oral biofilm and may lead to tooth loss. Periodontitis has been associated with various systemic diseases such as cardiovascular disease, adverse pregnancy outcomes and rheumatoid arthritis [1]. The global cost of this disease is not negligible and a better understanding of its etiopathogenicity could provide the key to its prevention and/or the improvement in its treatment.

The analysis of the periodontal biofilm revealed the presence of many different microorganisms including protozoans. A number of studies have evaluated the presence of protozoans in patients with periodontal diseases and the frequency of detection of *Entamoeba gingivalis* and *Trichomonas tenax* varied respectively from 12% to 69% and from 6% to 38.5% according to the authors [2–4]. After the discovery of genetic variation of E. gingivalis in immunocompromised patients by [5], a new subtype of *E. gingivalis* isolated from samples of patient with periodontitis has been recently identified and named kamaktli variant. The periodontal parameters associated with the presence of this subtype have not been described [5,6]. The differences in collection and identification protocols (microscopic observation/molecular tools), in the study population (adult/children, patients with or without systemic disease) and the inadequate characterization of periodontal diseases make it difficult to draw solid conclusions on the real prevalence of these microorganisms in periodontal disease. Moreover, no studies, except that of Linke’s [7], have detailed the periodontal pocket depth associated with the presence of these protozoans.

The Socransky modified Koch’s postulates are used to distinguish pathogenic microorganisms from non-pathogenic ones in periodontitis [8]. The postulate of ‘Association’ was partially satisfied by previous studies, even if the periodontal context in which these protozoans were found is rarely described [4]. The second postulate on ‘Elimination’ was advanced by Rashidi Maybodi [9] but did not associate the decrease of *T. tenax* with periodontal parameters. To date, the role of these protozoans in periodontitis remains unclear and requires further research.

This study is the first to evaluate the periodontal and bacterial context in which two *Entamoeba* subtypes and *T. tenax* have been identified, and the impact of scaling and root planing (SRP) on the prevalence of these protozoans in pathological and healthy sites in patients with periodontitis.

## Materials and methods

### Study design and setting

**Figure 1.**
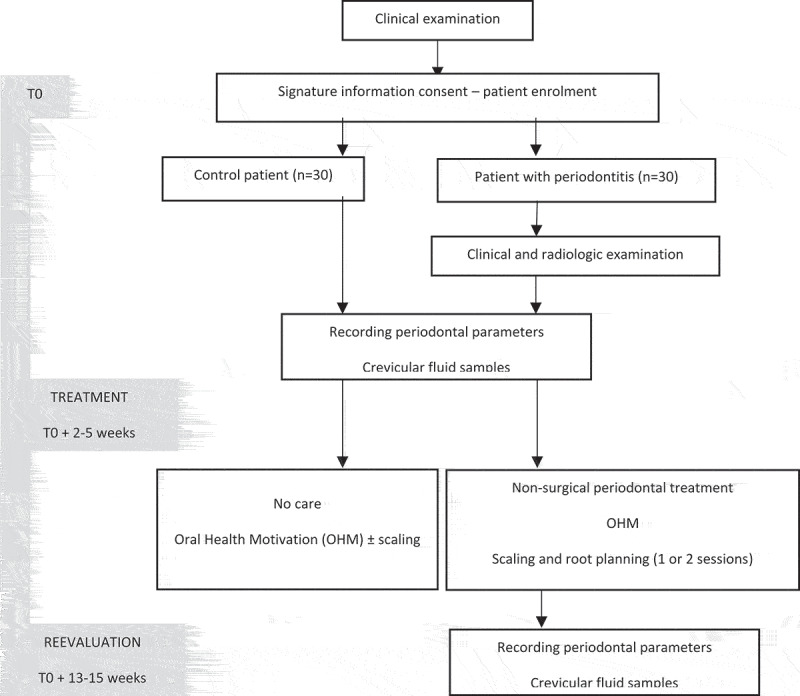
Design of the study

This monocentric clinical study was conducted in the periodontology department of the Nancy University Hospital between April 2016 and October 2017, was independently reviewed and approved by the Ethical Committee of Nancy University Hospital (Decision number: CPP/16/03/04) and was registered to the clinicaltrials.com website receiving the following number: NCT02873949. Protozoans were identified from samples collected as part of this study, Number DC-2016-2623, Ministry of Research, Paris, France. This is a comparative study [case-control (patients with vs without periodontitis)], a before-and-after case study (treatment), with a prospective extensive recruitment of both case and control eligible. The design of the study is provided in Figure 1.

### Study population

Patients with periodontitis (30 persons, including 17 women and 13 men, with a median age of 51.0 years, ranging from 38 to 65 years) were selected according to the following criteria: adult patients (≥18 years) (1) with stage II at IV and grade A to C periodontitis, according to the Chicago classification [10] (formerly chronic periodontitis, Armitage classification [11]) (2) with at least two periodontal pockets of 5 mm or more and one healthy site (probing depth ≤ 3 mm) (3) with no medication that could modify their immunity and oral microbiota (4) who had not received antibiotic therapy or root planing in the last 6 months and (5) pregnant women were excluded.

Moreover, 30 control patients, 17 women and 13 men with median age of 55.0 years, ranging from 22 to 70 years, were selected according to the following criteria: adult patients (1) without periodontal disease or systemic pathology (2) consulting for dental control or scaling.

### Data collection

The primary outcome is the evolution of the percentage of protozoans before and after SRP, and the secondary is the bacteria and periodontal parameters associated with their presence.

- Clinical periodontal parameters

All patients received a full periodontal examination and the following clinical periodontal parameters were recorded: (1) probing pocket depth (PPD) and (2) the clinical attachment level (CAL) using a William’s probe, (3) bleeding on probing (BOP), (4) tooth mobility, (5) the Plaque Index and (6) the Gingival Index [PlI [12], and GI [13]].

(ii) *Gingival Crevicular Fluid samples*

Two sites with a depth of 5 mm or more and one site with a depth of 3 mm or less in periodontitis patients and one healthy site (entitled control site in table and figure) from a control patient were selected. After cleaning and isolation of the selected tooth with a cotton pellet, gingival crevicular fluid (GCF) was collected using two sterile absorbent paper points for 30 s in the sulcus or pockets. The crevicular samples were placed in coded sterile tubes and stored at −80°C until analysis of the microbiota.

(iii) *Periodontal treatment*

All patients received oral hygiene instructions and patients diagnosed with periodontitis received SRP using ultrasound and/or manual curettes for the entire mouth (Figure 1).

The same practitioner assessed the similar periodontal parameters and collected, with a similar protocol, the crevicular samples (i) both before (baseline) and 2.5 months after the treatment (re-evaluation) in the same selected sites, without having the first record card during the re-evaluation, (ii) for both control and periodontitis patients.

### Microbial detection

- Extraction of microbial DNA

Microbial DNA was extracted and purified from paper points with the QIAamp DNA mini kit® (Qiagen, France) according to the manufacturer’s instructions. In summary, 400 µL of the AL buffer solution was added to microcentrifuge tubes, each containing two paper points from pathological, healthy or control sites. An incubation step (lysis) at 56°C for 30 min with 20 µL of Proteinase K followed by several washing steps. Finally, the DNA was resuspended in 75 µL of elution buffer.

(ii) *Bacteria PCR hybridization system*

Eleven periodontopathogens: *Porphyromonas gingivalis, Treponema denticola, Tannerella forsythia, Aggregatibacter actinomycetemcomitans, Prevotella intermedia, Parvimonas micra, Eubacterium nodatum, Fusobacterium nucleatum/periodonticum, Campylobacter rectus, Eikenella corrodens* and *Capnocytophaga* spp were researched. The amplification and hybridization of bacterial DNA were performed using the PCR system MicroIdent ®Plus11 kit (Hain LifeScience). Further details are provided in Supplementary data 1.

(iii) *Bacterial qPCR*

Total bacteria count was determined by a real-time PCR by the Clinident Institute for pathological sites (Aix-en-Provence, France). The minimum detection threshold was 1. 10 e4 and the minimum quantification threshold was 2. 10 e4 bacteria.

(iv) *Protozoan qPCR*

After DNA samples extraction by using the QIAamp DNA Mini Kit (Qiagen, France), TaqMan PCR amplifications were performed for each protozoan target. Briefly, the EG1F (5ʹ-TACCATACAAGGAATAGCTTTG-3ʹ) and EG2R (5ʹ-GATATTTTCATTGATTCCTTGTC-3ʹ) primers were designed to amplify a 153 bp fragment inside the SSU rDNA of *E. gingivalis*. The EG12P (5ʹ-FAM-GAATAGGCGCATTTCGAACAGGA-BHQ1) and EG12PK (5ʹ-HEX-AGTTGTTTGTACAAGTGGGCGCAT-BHQ1) TaqMan probes were respectively chosen to be specific of the *E. gingivalis* subtype 1 or 2 (var kamaktli) (ST1 and ST2). The TT5PS (5ʹ-GGAGTTGCATACATCATGAC-3ʹ), TT5PAS (5ʹ-CGTATAGCAGACAACGTAAGT-3ʹ) primers and the TT5S (5ʹ-HEX-CTAAACTTGGCTTCGGCTGAGAA-BHQ2) TaqMan probe were also designed to specifically amplify a 5.8 S rDNA fragment of *Trichomonas tenax*.

The validity of these TaqMan PCR assays was assessed through the study of several criteria: specificity, repeatability, reproducibility, cross-contamination risk, efficiency and analytical sensitivity (LOD = limit of detection). These data were previously described [14]. The detailed protocol is described in Supplementary data 2.

### Statistical analysis

According to previous data published [4], we considered that *T. tenax* and *E. gingivalis* are found in about 38.5% and 54.8% respectively of periodontitis patients. Our hypothesis is that SRP is effective to eliminate 80% of these protozoans as shown for some periodontopathogens, with a prevalence after treatment estimated at 5%. Considering an α risk of 5% and a power of 80%, it was then necessary to include 30 periodontitis patients to show a significant difference in the protozoan prevalence between and after treatment (Power and Sample Size Calculation, version 3.1.2). We planned to include the same number of control patients to maximize the statistical power of comparisons planned between the case and control groups.

Clinical and microbial characteristics were then compared between (i) control sites and healthy sites of periodontitis patients before treatment, (ii) healthy sites of periodontitis patients before and after treatment, (iii) pathological sites before and after treatment, by using the Fisher’s or McNemar test as appropriate for categorical variables and the Wilcoxon’s test for continuous variables. Finally, for periodontitis group before treatment, demographic, clinical and microbial parameters of pathological sites associated with the presence of each parasite (*E. gingivalis* ST1, ST2, *T. tenax*) were identified using Fisher and Wilcoxon’s tests. Significance levels were fixed at p ≤ 0.05, and two-sided tests were used. All analyses were performed using SAS version 9.4 (SAS Institute, Inc., Cary, NC, USA). More statistical details are provided in Supplementary data 3.

## Results

### Population study

The demographic data of both groups are summarized in Table 1. Age and female/male percentage are similar between the control group and the periodontitis group (55.0 vs. 51.0 years and 56.7% (N = 17)/43.3% (N = 13) vs 56.7% (N = 17)/43.3% (N = 13) respectively, p > 0.05). 43.3% of periodontitis patients are smokers compared to only 16.7% of control patients (p = 0.02).

**Table 1.** Demographic characteristics and protozoan positivity of studied population

	Control patients (n = 30)				Periodontitis patients (n = 30)			
Demographic information	N	%	Med[Min-Max]		N	%	Med[Min-Max]	p*
Female sex	17	56.7			17	56.7		1.00
Age (years)	30		55.0 [22–70]		30		51.0 [38–65]	0.91
Non-Smoker	25	83.3			17	56.7		**0.02**
				Before treatment#		After treatment#		
Patient protozoan positivity	N	%		N	%	N	%	p*
Protozoan positivity¤	1	3.3		28	93.3	26	86.7	**<0.001**
*Association ST1/ST2*	0	0		5	16.7	1	3.3	-
*Association ST1/T. tenax*	0	0		10	33.3	6	20	-
*Association ST2/T. tenax*	0	0		2	6.67	1	3.3	-

*Notes*: The comparisons of values were assessed by the exact McNemar test or symmetry test for qualitative variables and the Wilcoxon test for quantitative variables. * comparison between control and periodontitis patients before treatment. A p-value of less than 0.05 is considered significant. ¤ Protozoan positivity for *E. gingivalis* ST1 + *E. gingivalis* ST2 + *T. tenax*. # in pathological sites

### Before non-surgical treatment

- Clinical periodontal parameters

Clinical periodontal parameters were recorded prior to treatment for each selected site of both groups (Table 2). The pathological sites presented a median PPD and CAL of respectively 7 mm and 8 mm. Among the diseased sites, 55% of sampled teeth are mobile and 60% bleed on probing.

(ii) *Microbiota*

- Protozoans

**Table 2.** Clinical parameters and microbial positivity of samples from patients of periodontitis and control group before and after SRP treatment

					Periodontitis patients (n = 30)														
		Control patients (n = 30)			Healthy sites (n = 30)								Pathological sites (n = 60)						
		Control sites* (n = 30)			Before treatment#*			After treatment#					Before treatment°			After treatment°			
	Grades	N	%	Med [Min-Max]	N	%	Med [Min-Max]	N	%	Med [Min-Max]	p#	p*	N	%	Med [Min-Max]	N	%	Med [Min-Max]	p°
**Clinical characteristics**																			
Plaque Index	0	27	90		24	80		27	90		0.39	0.40	19	31.7		30	50.0		0.11
	1	3	10		4	13		3	10				22	36.7		21	35.0		
	2	0	0		2	6.7		0	0				16	26.7		7	11.7		
	3	0	0		0	0		0	0				3	5.0		2	3.3		
Gingival Index	0	28	93.3		13	43.3		23	76.7		**0.01**	**<0.001**	0	0		16	26.7		**<0.001**
	1	2	6.7		16	53.3		6	20				25	41.7		33	55.0		
	2	0	0		1	3.3		1	3.3				26	43.3		9	15.0		
	3	0	0		0	0		0	0				9	15.0		2	3.3		
PPD (mm)		30		1.0 [1.0–3.0]	30		2.0 [1.0–3.0]	30		1.0 [1.0–3.0]	0.59	0.34	60		7.0 [5.0–11]	60		5.0 [2.0–13]	**<0.001**
CAL (mm)		30		1.5 [1.0–3.0]	30		2.0 [1.0–5.0]	30		2.0 [1.0–6.0]	1.00	0.07	60		8.0 [5.0–16]	60		6.0 [3.0–20]	**<0.001**
Mobile teeth		0	0		1	3.3		1	3.3		1.00	1.00	33	55		36	60		0.51
BOP		0	0		0	0		0	0		1.00	1.00	36	60		29	48.3		0.25
**Microbial positivity in samples**																			
*E. gingivalis ST1*		1	3.3		3	10		4	13.3		1.00	0.61	42	70		35	58.3		0.06
*E. gingivalis ST2*		0	0		1	3.3		0	0		1.00	-	11	18.3		8	13.3		0.37
*Trichomonas tenax*		0	0		2	6.7		2	6.7		0.50	1.00	20	33.3		11	18.3		**0.004**
**Associations**																			
*ST1/ST2*		0	0		0	0		0	0		-	-	0	0		1	1.7		-
*ST1/T. tenax*		0	0		1	3.3		1	3.3		-	-	17	28.3		10	16.7		-
*ST2/T.tenax*		0	0		0	0		0	0		-	-	2	3.3		1	1.7		-
**Total bacterial count** (10 e7)			nd			nd			nd		nd	nd	60		6.0 [3.1–9.8]	60		1.9 [5.8–5.7]	**<0.001**

Note: PPD: periodontal pocket depth; CAL: Clinical attachment level; BOP: Bleeding on probing

The comparisons of values were assessed by the exact McNemar test or symmetry test for qualitative variables and the Wilcoxon test for quantitative variables. # comparison between before and after treatment in healthy sites; * comparison between healthy and control sites before treatment; ° comparison between before and after treatment in pathological sites. A p-value of less than 0.05 was considered significant.

**Figure 2.**
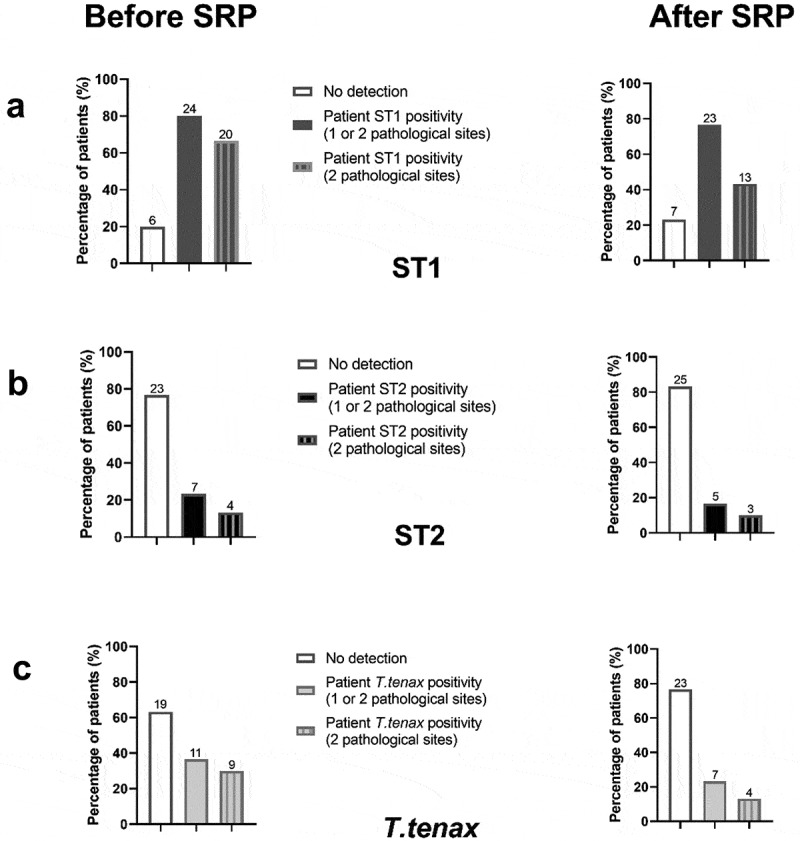
Protozoans positivity in periodontitis patients according to the number of pathological sites concerned before and after the SRP. (a) *E. gingivalis* ST1, (b) *E. gingivalis* ST2, (c) *T. tenax.*

The protozoan positive status of patients in the control and periodontitis group is described in Table 1 and potential association of demographic data with each amoeba positivity are provided in Table 3. No association between demographic data and protozoan’s positivity was found (p > 0.05). As regards *E. gingivalis* ST1 patient positivity, 80% of periodontitis patients presented this amoeba in one or two and only 66.7% in two pathological sites (n = 24 and n = 20, respectively) (Figure 2(a)). Concerning *E. gingivalis* ST2 and *T. tenax* patient positivity, 7 periodontitis patients (23.3%) presented ST2 and 11 (36.7%) *T. tenax* in one or two pathological site, and only 13.3% (n = 4) and 30% (n = 9) of them in both periodontal sites, respectively (Figure 2(b,c)). Moreover, regarding the co-localisation of ST1 and *T. tenax* in periodontitis patients, 33.3% of them presented both ST1 and *T. tenax* in at least one of their pathological sites. The association ST1 and ST2 concerned five patients (16%) while the association of ST2 and *T. tenax* concerned only two patients (6.7%) (Table 1).

**Table 3.** Demographic data from periodontitis group according to the *E. gingivalis* ST1, *E. gingivalis* ST2 and *T. tenax* positivity before periodontal treatment

		Female sex			Age (years)			Non-smoker		
		N	%	p	N	Med [Min-Max]	p	N	%	P
*E. gingivalis* ST1										
	Yes	12	50.0	0.20	24	51.0 [38–65]	0.90	12	50.0	0.20
	No	5	83.3		6	53.5 [40–64]		5	83.3	
*E. gingivalis* ST2										
	Yes	5	71.4	0.43	7	58.0[40–64]	1.00	6	85.7	0.10
	No	12	52.2		23	51.0 [38–65]		11	47.8	
*T. tenax*										
	Yes	6	54.5	1.00	11	51.0 [40–63]	0.67	8	72.7	0.26
	No	11	57.9		19	51.0 [38–65]		9	47.4	

*Notes*: Chi-2 or Fisher exact tests were used for the statistical analysis of qualitative variables (Gender and smoking status) and Wilcoxon test for quantitative variables (Age). A p-value of less than 0.05 is considered significant.

The values are expressed in percentage. The numbers above the columns correspond to the number of patients concerned.

The protozoan positive status of samples in the periodontitis group is described in Table 2. *T. tenax, E. gingivalis* ST1 and ST2 were, respectively, identified in two, three and one healthy site. Among the pathological sites, before treatment none presented ST1 and ST2 at the same time and in 40.5% of them (n = 17) ST1 and *T. tenax* were co-identified from the same site. Only one healthy site from the control group was positive for ST1.

- Bacteria

**Figure 3.**
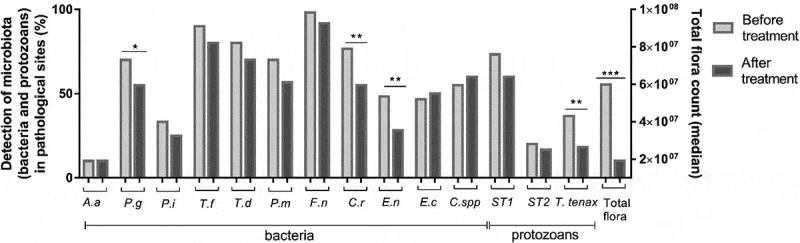
Microbiote’s evolution after treatment

Among the 11 bacteria detected, six were highly present in 70% or more of the pathological sites before periodontal treatment: *P. gingivalis, T. forsythia, T. denticola, P. micra, F. nucleatum* and *C. rectus* (Figure 3).

(iii) *Clinical and bacterial parameters associated with the presence of each parasite*

The values of each bacterium or protozoans are expressed in percentage and the total flora count are expressed in median. The McNemar test was used to compare the percentage of microbiota before and after the treatment and the median of the total flora count. *p < 0.05, **p < 0.01, ***p < 0.001

Concerning *E. gingivalis* ST1: in the bivariate analysis, the presence of ST1 in the pathological sites was correlated only with GI and CAL (p < 0.01 and p = 0.03, respectively). Among pathological sites ST1 positive, 26.2% of ST1 was found in 5–6 mm PPD and 73.8% in ≥7 mm PPD, which represents a protozoan positivity of 55% and 77.5% of each PPD class, respectively. Concerning the bacteria, ST1 was significantly associated with a high level of total bacterial load (p = 0.02), but not with any particular bacteria (Table 4).

**Table 4.** Clinical and microbial parameters of pathological sites from the periodontitis group according to the *E. gingivalis* ST1 positivity in samples before periodontal treatment

		Positivity of *Entamoeba gingivalis* ST1						
		No			Yes			
		N	%	Med [Min-Max]	N	%	Med [Min-Max]	
	Grades	18	30.0		42	70.0		p
**Clinical parameters**								
Plaque Index	0	7	38.9		12	28.6		0.79
	1	6	33.3		16	38.1		
	2	5	27.8		11	26.2		
	3	0	0.0		3	7.1		
Gingival Index	1	12	66.7		13	31.0		**0.01**
	2	6	33.3		20	47.6		
	3	0	0		9	21.4		
PPD	5–6 mm	9	50.0		11	26.2		0.07
	≥7 mm	9	50.0		31	73.8		
CAL (mm)		18		7.0 [5.0–11]	42		8.5 [5.0–16]	**0.03**
Mobile teeth		11	14.9		23	50.0		0.95
BOP		10	55.6		26	61.9		0.65
**Microbial association*- With protozoan***								
*E. gingivalis* (ST2)		11	61.1		0	0		**<0.001**
*T. tenax*		3	16.7		17	40.5		0.07
***- With bacteria***								
*A.a*		3	16.7		3	7.1		0.35
*P.g*		11	61.1		31	73.8		0.32
*P.i*		6	33.3		14	33.3		1.00
*T.f*		16	88.9		38	90.5		1.00
*T.d*		12	66.7		36	85.7		0.16
*P.m*		12	66.7		30	71.4		0.71
*F.n*		17	94.4		42	100		0.30
*C.r*		14	77.8		32	76.2		1.00
*E.n*		8	44.4		21	50.0		0.69
*E.c*		9	50.0		19	45.2		0.73
*C*.spp		13	72.2		20	47.6		0.08
Total bacterial load (10 e7)		18		3.39 [0.19–20.0]	42		6.43 [0.13–54.0]	**0.02**

*Note: PPD: periodontal pocket depth; CAL: clinical attachment level; BOP: bleeding on probing; A.a: Aggregatibacter actinomycetemcomitans; P.g: Porphyromonas gingivalis; P.i: Prevotella intermedia; T.f: Tannerella forsythia; T.d: Treponema denticola; P.m: Parvimonas micra; F.n: Fusobacterium nucleatum; C.r: Campylobacter rectus; E.n: Eubacterium nodatum; E.c: Eikeinella corrodens; Cs*pp*: Capnocytophaga* species; Total bacterial load: bacterial number for 1µl DNA.

The exact Fisher test was used for the statistical analysis of qualitative variables and the Wilcoxon test for quantitative variables (CAL and total bacterial load). A p-value of less than 0.05 was considered significant.

Concerning *E. gingivalis* ST2: in the bivariate analysis, the presence of ST2 was not associated with the ST1 or *T. tenax* presence or any clinical periodontal parameters except for PPD. Indeed, among pathological sites ST2 positive, 63.6% of ST2 was found in 5–6 mm PPD and 36.4% in ≥7 mm PPD, which represent a protozoan positivity of 35% and 10% of each PPD class, respectively (p = 0.03). Its association with *C. rectus* is almost significant (p = 0.05) (Table 5).

**Table 5.** Clinical and microbial parameters of pathological sites from the periodontitis group according to the *E. gingivalis* ST2 positivity in samples before periodontal treatment

		Positivity of *Entamoeba gingivalis* ST2						
		No			Yes			
		N	%	Med [Min-Max]	N	%	Med [Min-Max]	
	Grades	49	81.7		11	18.3		p
**Clinical parameters**								
Plaque Index	0	17	34.7		2	18.2		0.45
	1	18	36.7		4	36.4		
	2	11	22.4		5	45.5		
	3	3	6.1		0	0.0		
Gingival Index	1	19	38.8		6	54.5		0.33
	2	21	42.9		5	45.5		
	3	9	18.4		0	0.0		
PPD	5–6mm	13	26.5		7	63.6		**0.03**
	≥7 mm	36	73.5		4	36.4		
CAL (mm)		49		8.0 [5.0–16.0]	11	7.0	7.0 [5.0–11.0]	0.07
Mobile teeth		24	14.9		9	81.8		0.09
BOP		32	65.3		4	36.4		0.10
**Microbial association**								
***- With protozoans***								
*E. gingivalis* (ST1)		42	85.7		0	0		**<0.0001**
*T. tenax*		18	36.7		2	18.2		0.31
***- With bacteria***								
*A.a*		6	12.2		0	0		0.58
*P.g*		35	71.4		7	63.6		0.72
*P.i*		14	28.6		6	54.5		0.16
*T.f*		43	87.8		11	100		0.58
*T.d*		37	75.5		11	100		0.10
*P.m*		33	67.3		9	81.8		0.48
*F.n*		48	98.0		11	100		1.00
*C.r*		35	71.4		11	100		0.05
*E.n*		21	42.9		8	72.7		0.07
*E.c*		23	46.9		5	45.5		0.93
*C*.spp		24	49.0		9	81.8		0.09
Total bacterial load (10 e7)		49		6.1 [0.13–54.0]	11	3.94	3.94 [0.43–20.1]	0.28

*Note: PPD: periodontal pocket depth; CAL: clinical attachment level; BOP: bleeding on probing; A.a: Aggregatibacter actinomycetemcomitans; P.g: Porphyromonas gingivalis; P.i: Prevotella intermedia; T.f: Tannerella forsythia; T.d: Treponema denticola; P.m: Parvimonas micra; F.n: Fusobacterium nucleatum; C.r: Campylobacter rectus; E.n: Eubacterium nodatum; E.c: Eikeinella corrodens; C*spp*: Capnocytophaga* species; Total bacterial load: bacterial number for 1µl DNA.

The exact Fisher test was used for the statistical analysis of qualitative variables and the Wilcoxon test for quantitative variables (CAL and total bacterial load). A p-value of less than 0.05 was considered significant.

Concerning *T. tenax*: none of the clinical periodontal parameters were statistically linked to the presence of *T. tenax* in the bivariate analysis, in pathological sites in the periodontitis group. However, in pathological samples, this flagellate was statistically correlated with higher total sub-gingival flora (p = 0.01) and with some bacteria: *P. gingivalis, T. denticola* and *E. nodatum* (p = 0.02, p = 0.005, p = 0.02, respectively) (Table 6).

**Table 6.** Clinical and microbial parameters of pathological sites from the periodontitis group according to the *T. tenax* positivity in samples before periodontal treatment

		*Positivity of Trichomonas tenax*						
		No			Yes			
		N	%	Med [Min-Max]	N	%	Med [Min-Max]	
	Grades	40	66.7		20	33.3		
**Clinical Parameters**								
Plaque Index	0	13	32.5		6	30.0		1.00
	1	14	35.0		8	40.0		
	2	11	27.5		5	25.0		
	3	2	5.0		1	5.0		
Gingival Index	1	18	45.0		7	35.0		0.81
	2	16	40.0		10	50.0		
	3	6	15.0		3	15.0		
PPD	5–6 mm	15	37.5		5	25.0		0.33
	≥7 mm	25	62.5		15	75.0		
CAL (mm)		40		8.0 [5.0–11.0]	20		9.0 [6.0–16.0]	0.17
Mobile teeth		22	55.0		11	55.0		1.00
BOP		23	57.5		13	65.0		0.58
**Microbial association**								
***- With protozoans***								
*E. gingivalis* (ST1)		25	62.5		17	85.0		0.07
*E.gingivalis* (ST2)		9	22.5		2	10.0		0.31
***- With bacteria***								
*A.a*		6	15.0		0	0		0.17
*P.g*		24	60.0		18	90.0		**0.02**
*P.i*		14	35.0		6	30.0		0.70
*T.f*		36	90.0		18	90.0		1.00
*T.d*		28	70.0		20	100		**0.005**
*P.m*		27	67.5		15	75.0		0.55
*F.n*		39	97.5		20	100		1.00
*C.r*		28	70.0		18	90.0		0.11
*E.n*		15	37.5		14	70.0		**0.02**
*E.c*		17	42.5		11	55.0		0.36
*C*.spp		21	52.5		12	60.0		0.58
Total bacterial load (10 e7)		40	5.13	5.13 [0.13–54.0]	20	8.55	8.55 [0.71–21.8]	**0.01**

*Note: PPD: periodontal pocket depth; CAL: clinical attachment level; BOP: bleeding on probing; A.a: Aggregatibacter actinomycetemcomitans; P.g: Porphyromonas gingivalis; P.i: Prevotella intermedia; T.f: Tannerella forsythia; T.d: Treponema denticola; P.m: Parvimonas micra; F.n: Fusobacterium nucleatum; C.r: Campylobacter rectus; E.n: Eubacterium nodatum; E.c: Eikeinella corrodens; C*spp*: Capnocytophaga* specie*s*; Total bacterial load: bacterial number for 1µl DNA.

The exact Fisher test was used for the statistical analysis of qualitative variables and the Wilcoxon test for quantitative variables (CAL and total bacterial load). A p-value of less than 0.05 was considered significant.

### After non-surgical treatment

- Clinical periodontal parameters

The pathological sites from periodontitis patients presented a significant decrease in many clinical parameters: distribution of GI, PPD, CAL (p < 0.001). Concerning the healthy sites from the periodontitis group, only gingival inflammation was significantly reduced (p = 0.01) (Table 2).

- Microbiota

- Protozoans

About the pathological sites in patients with periodontitis, only *T. tenax* displays a significant decrease in presence after SRP, with absolute and relative reduction of 11% and 50% respectively (p = 0.001). Moreover, *T. tenax* was found in a lower quantity after SRP (increase in the cycle threshold of PCR- Supplementary Table 1). After periodontal treatment, some healthy sites from the periodontitis group experienced ST1 elimination, others an occurrence or no modification of this amoeba presence. In addition, among the two *T. tenax*-positive healthy sites before therapy, one site remained positive and the other presented a flagellate elimination after treatment. Moreover, one healthy site negative before SRP become positive after. These variations of protozoan presence observed in healthy sites from periodontitis patients were not statistically significant (Table 2). The co-identification of both ST1 and ST2 was statistically rare and concerned one same sample alone (p < 0.0001).

Neither the protozoan presence nor their decrease was statistically associated with the improvement or the complete healing of the diseased sites (decrease of PPD or absence of pocket) (Supplementary Table 2).

- Bacteria

After the periodontal treatment, the detection of *P. gingivalis, C. rectus* and *E. nodatum* decreased significantly and a statistical trend was observed with *T. forsythia* (p = 0.07) (Figure 3). Moreover, a significant and marked reduction in the total microbial count was also observed (p < 0.001).

## Discussion

This study shows the presence of two *E. gingivalis* subtypes and *T. tenax* in subgingival biofilm from pathological sites, and more rarely from healthy sites of patients with periodontitis. Only one control patient presented *E. gingivalis* ST1. The SRP induced a significant decrease of *T. tenax* but not of *E. gingivalis* ST1 and ST2, and neither the decrease and/or absence of these parasites was significantly associated with PPD improvement of pathological sites.

Even if recent advances in technology provide us new insights into periodontitis etiopathogenesis with the dysbiosis concept and the highlighting of complex interactions of bacteria inside a structured biofilm, the Koch’s postulate revisited by Socransky remains an attractive tool to evaluate the pathogenicity of microorganisms [15].

In this study, the frequency of detection of *E. gingivalis* in subgingival biofilm samples from the periodontal pockets of patients without systemic disease (ST1 or ST2: 88.3%) is higher than in most previous studies [2,16] using molecular tools such as Trim et al. (69%) [2]. These differences are certainly linked to (i) the microbiota collection protocol [number of paper points used for the collection, localisation of the sampled microbiota (supra or sub-gingival), contamination of the sub-gingival sample with saliva and/or supra-gingival biofilm], (ii) inadequate parameters used to define periodontitis (for some authors, the patient included presented at least two of the following criteria: oedema, bleeding on probing, gum recession, tooth mobility and a pocket depth ≥ 3 mm which could be, with the exclusion of the periodontal pocket, the clinical parameters of both gingivitis and periodontitis) (iv) at the targeted subtype. Garcia et al. [17] have investigated the presence of both *E. gingivalis* ST1 and ST2 in the oral cavity and found a higher percentage of healthy patients positive to both ST1 and ST2 and patient with periodontal disease to ST2, but they observed a lower positivity for ST1 in the periodontal group than our results [17]. These discrepancies could be explained, as already mentioned above, by the different protocols used [sample collection, PCR methodology (amoeba detection by nested PCR analysis)].

Concerning *T. tenax*, only one study using molecular tools evaluated the frequency of this protozoan in biofilm from patients with periodontitis, but the study population had Down syndrome and could not be compared with our results [18]. However, the prevalence of *T. tenax* in our study is consistent with previous publications that have detected this protozoan under microscopy [4].

In previous publications, the periodontal and bacterial context associated with the presence of protozoans is very rarely described. Concerning *E. gingivalis*, the authors have observed a higher prevalence of this amoeba in their patients with a 4–6 mm PPD than in those with a 7–9 mm PPD, 53% and 18%, respectively, [7]. As these authors detected this protozoan through microscope observation, they were unable to differentiate between *E. gingivalis* ST1 and ST2. In our study, among all the periodontal pockets *E. gingivalis* positive, ST1 seems to be more frequently found in 7 mm PPD and above than in 5–6 mm (p = 0.07), and the opposite was observed for ST2 (p = 0.03). ST1 was significantly associated with gingival edema and a high total bacterial flora from subgingival biofilm samples. Certainly, amoebae need actively dividing bacteria to provide them with a favourable environment for their growth. Gannon and Linke [19] demonstrated that the diffusion of *Yersinia enterocolitica* cellular metabolites in the amoeba growth chamber increased its growth. Surprisingly, this study did not find any particular bacterial association with ST1 although various authors described the simultaneous presence of *E. gingivalis* and *Actinomyces, E. corrodens* and *Prevotella* sp. in an extra-oral localization such as osteomyelitis and brain abscess [20,21]. These well-known periodontal bacteria identified in extra-oral localization may develop different bacterial interactions than those found in periodontal biofilm due to a less complex bacterial composition.

The lack of association of protozoans with a high amount of supragingival plaque could be due to differences in bacterial composition and biochemical environment (ae/anaerobic condition, pH, temperature) between supra-gingival and sub-gingival biofilms that are not suitable for amoebic proliferation [22]. The ST2 protozoan is not correlated to any clinical characteristics (excluding PPD) or parameters and seems to be randomly found in some pathological sites. ST1 and ST2 amoeba were found together in only one sample out of 120. These two amoebae could be in competition for a nutrient, which could explain their lack of presence in the same pathological site, as seen between two strains of malaria parasites in vivo [23].

The flagellate is statistically correlated with a high amount of total bacterial flora and with different bacteria such as *P. gingivalis, T. denticola* and *E. nodatum*. The co-identification of both *T. tenax* and some periodontopathogens in the same site is not surprising as both inhabit oxygen-poor environments [24] Nutrient co-operation could certainly occur within the biofilm between these microorganisms as demonstrated between *A. actinomycetemcomitans* and unidentified oral amoebae [25].

As expected, SRP improved many clinical and microbial parameters. Only *T. tenax* was statistically reduced in subgingival biofilm. Rashidi Maybodi [9] found a significant reduction of amoeba in both saliva and dental plaque, and only in saliva for flagellates [9]. The lack of a precise location of the sampled plaque and the detection of protozoan under the microscope could explain the discrepancy with our results. A significant decrease in bacterial load and in the detection of some periodontopathogens was observed. As previously described, this bacterial decrease was correlated with clinical improvement, but in our study, neither the elimination nor the reduction of the presence of protozoan was associated with healing and/or improvement of the PPD parameter [26].

Protozoans could have an indirect role in the development of periodontitis. Both amoebae and flagellates presented intracytoplasmic bacteria which could be alive or dead inside large food vacuoles [27,28] An *in vitro* study showed the capacity of two periodontal pathogens, *P. gingivalis* and *P. intermedia*, to enter, survive and replicate in *Acanthamoeba castellanii*, a protozoan identified in the oral cavity [29,30]. These results suggest that amoeba could be a reservoir for these bacteria and protect them from immune cells and antibiotics such as amoxicillin and participate in the re-colonization of periodontal pockets after non-surgical treatment.

This original study presents preliminary results and could be improved by a multicentric recruitment of patients to avoid potential selection bias and a higher sample size of patients with periodontitis which would enable us to evaluate a larger periodontal clinical characteristic, a possible reduction of *E. gingivalis* after SRP treatment and bacterial composition in order to clearly identify some factors possibly associated with protozoan colonization.

This is the first study to identify two subtypes of amoebae and *T. tenax* in subgingival microbiota, their association with total bacterial load (excluding ST2) and certain periodontal parameters and to demonstrate that amoeba were relatively unaffected by non-surgical periodontal treatment. *T. tenax* were statistically correlated with many major periodontopathogens. The co-identification of protozoans and bacteria in the same pocket sample requires further investigation study for a better understanding of their interactions. To date, the pathogenicity of both *E. gingivalis* and *T. tenax* in periodontitis is still debated and requires further study.

## Supplementary Material

Supplemental Material
