# Supplementary material for: Protozoans in subgingival biofilm: clinical and bacterial associated factors and impact of scaling and root planing treatment
Source: J Oral Microbiol. 2019 Nov 25;12(1):1693222. doi: 10.1080/20002297.2019.1693222 (PMC6882485; doi:10.1080/20002297.2019.1693222)
Supplement: Supplemental Material [file ZJOM_A_1693222_SM6097.docx]

**Protozoans in subgingival biofilm: clinical and bacterial associated factors and impact of scaling and root planing treatment.**

**Supplementary files**

**Supplementary data 1. Microbial detection** - *Bacteria PCR hybridization system*

The purity and quantity of DNA were assessed using the spectrometer Nanodrop™ 2000C (ThermoScientific). The minimum detection threshold of this kit is 10^4^ bacteria in the sample except for *A. actinomycetemcomtans*: 10^3^. The interpretation of the colorimetric bands’ intensity was carried out by two independent individuals. The accuracy of the PCR system MicroIdent®*Plus*11 kit has been tested by some authors (Santigli et al., 2016, Haffajee et al., 2009).

References:

Haffajee, A. D., Yaskell, T., Torresyap, G., Teles, R., Socransky, S. S. (2009). Comparison between polymerase chain reaction-based and checkerboard DNA hybridization techniques for microbial assessment of subgingival plaque samples. *Journal of Clinical Periodontology*, 36, 642‑649.

Santigli, E., Leitner, E., Wimmer, G., Kessler, H. H., Feierl, G., Grube, M., et al. (2016). Accuracy of commercial kits and published primer pairs for the detection of periodontopathogens. *Clinical Oral Investigations*, 20, 2515‑2528.

**Supplementary data 2**. **Microbial detection** - *Protozoan qPCR*

- *Choice of primers and probes*

The choice of primers was made according to a method described in another article that recently has been accepted in the journal "Diagnostic Microbiology & Infectious Disease" (Machouart et al., 2019).

In summary, a bioinformatics analysis was conducted with the 30 *Entamoeba* sp. SSU rDNA following sequences available on GenBank (<https://www.ncbi.nlm.nih.gov/genbank/>), and corresponding to these species : 5 *E. gingivalis* subtype 1 (KF250435; KF250436; KF250434; KF250433; D28490), 7 *E. gingivalis* subtype 2 corresponding to the kamaktli variant (KX027286; KX027287; KX027288; KX027289; KX027294; KX027295; KX027296), 4 *E. coli* (FR686364; AB444953), 1 *E. moshkovskii* (AF149906), 4 *E. histolytica* (GQ423750; GQ423749; GQ423748; AB426549), 1 *E. dispar* (AB282661), and 8 *E. polecki* (FR686400; FR686398; FR686397; FR686395; FR686394; FR686393; FR686392; FR686357).

Firstly, an alignment was made within each group of sequences corresponding to the same species, with the CLUSTAL Omega multiple sequence alignment program (<https://www.ebi.ac.uk/Tools/msa/clustalo/>), in order to select one representative sequence after controlling their similarity. Secondly, an alignment was made with the 7 selected sequences: *E. gingivalis* ST1 D28490, *E. gingivalis* ST2 KX027296; *E. histolytica* AB426549, *E. moshkovkii* AF149906, *E. dispar* Z49256, *E. coli* AB444953, *E. polecki* AF149913. The sequences of *T. vaginalis* JX943583, *T. foetus* M81842, *Candida albicans* M60302 and *C. tropicalis* M60308 were added to this alignment. Similarly, specificity was checked on different species of Trichomonas whose sequences were obtained from databases, but also with different bacterial and fungal species.

The bioinformatics specificity of each primers and probes were controlled with a systematic search of the corresponding sequences on the Nucleotide Basic Local Alignment Search Tool (BLAST : <https://blast.ncbi.nlm.nih.gov/Blast.cgi>).

- *The TaqMan PCR assays*

The TaqMan PCR assays were performed by using the QuantiTect Probe PCR kit (Qiagen, France). Each 50 µL reaction consisted of 25 µL 2X QuantiTect Probe PCR Master Mix, 1 µL of each primer (20 µM; 0.4 µM in final concentration), 0.5 µL of probe (20 µM in total; 0.2 µM in final concentration), and 17.5 µl of RNase-free water. Five microliters of patient DNA were added to each reaction. For all PCR reactions, an iCycler thermocycler equipped with a MyiQ2 Optics Module was used (version 2.1) under the following conditions: 95°C for 15 min of Taq polymerase activation; 45 cycles of a denaturation step at 94°C for 15 sec, followed by a combined hybridization and elongation at 60°C for 60 sec.

**Supplementary data 3.** **Statistical analysis**

Descriptive statistics were first used to assess demographic, clinical and microbial characteristics of patients from control and periodontitis groups. Continuous variables are presented as medians and interquartile intervals, and categorical variables as percentages.

**Supplementary tables**

**Supplementary Table 1**

**Supplementary TABLE 1.** Comparison of the number of cycle threshold of the protozoans before and after the periodontal therapy.

|  | **Before therapy** | | | **After therapy** | | |  |
| --- | --- | --- | --- | --- | --- | --- | --- |
|  | **N** | **Med** | **Min-Max** | **N** | **Med** | **Min-Max** | **p** |
| **Cycle treshold *E. gingivalis* ST1** | 42 | 28.13 | 24.66-36.11 | 35 | 30,91 | 26.70-36.32 | 0.99 |
| **Cycle threshold *E. gingivalis* ST2** | 11 | 30.85 | 28.21-35.24 | 8 | 30,17 | 28.03-34.74 | 0.15 |
| **Cycle threshold *T. tenax*** | 20 | 32.00 | 25.37-39.55 | 11 | 32,74 | 26.82-38.28 | **0.007** |

The comparisons of the values were assessed by the Wilcoxon test of the signed ranks. A p-value of less than 0.05 is considered significant.

**Supplementary Table 2**

**Supplementary TABLE 2.** Evolution of the pocket depth according to the presence or not of the different parasites.

|  | **Improvement of pathological sites**  **(n=50)** | | **No improvement of pathological sites**  **(n=10)** | |  |
| --- | --- | --- | --- | --- | --- |
|  | **N** | **%** | **N** | **%** | **p** |
| **Evolution of the presence of *E. gingivalis* ST1** |  |  |  |  |  |
| Absent before/absent after treatment | 13 | 26.0 | 3 | 30.0 | 0.62 |
| Absent before/present after treatment | 2 | 4.0 | 0 | 0.0 |  |
| Present before/absent after treatment | 9 | 18.0 | 0 | 0.0 |  |
| Present before/present after treatment | 26 | 52.0 | 7 | 70.0 |  |
| **Evolution of the presence of *E. gingivalis* ST2** |  |  |  |  |  |
| Absent before/absent after treatment | 41 | 82.0 | 7 | 70.0 | 0.27 |
| Absent before/present after treatment | 1 | 2.0 | 0 | 0.0 |  |
| Present before/absent after treatment | 4 | 8.0 | 0 | 0.0 |  |
| Present before/present after treatment | 4 | 8.0 | 3 | 30.0 |  |
| **Evolution of the presence of *T. tenax*** |  |  |  |  |  |
| Absent before/absent after treatment | 33 | 66.0 | 7 | 70.0 | 0.35 |
| Present before/absent after treatment | 9 | 18.0 | 0 | 0.0 |  |
| Present before/present after treatment | 8 | 16.0 | 3 | 30.0 |  |

The comparisons of the values were assessed by the Fisher’s exact test. A p-value of less than 0.05 is considered significant.
